# Does Rhizobial Inoculation Change the Microbial Community in Field Soils? A‍ ‍Comparison with Agricultural Land-use Changes

**DOI:** 10.1264/jsme2.ME24006

**Published:** 2024-09-12

**Authors:** Shintaro Hara, Kaori Kakizaki, Masaru Bamba, Manabu Itakura, Masayuki Sugawara, Atsuo Suzuki, Yuma Sasaki, Masanori Takeda, Kanako Tago, Tsubasa Ohbayashi, Toshihiro Aono, Luciano Nobuhiro Aoyagi, Hiroaki Shimada, Ryo Shingubara, Sachiko Masuda, Arisa Shibata, Ken Shirasu, Rota Wagai, Hiroko Akiyama, Shusei Sato, Kiwamu Minamisawa

**Affiliations:** 1 Institute for Agro-Environmental Sciences (NIAES), National Agriculture and Food Research Organization (NARO), Tsukuba, 305–8604, Japan; 2 Graduate School of Life Sciences, Tohoku University, Sendai, 980–8577, Japan; 3 Department of Life and Food Sciences, Obihiro University of Agriculture and Veterinary Medicine, Obihiro, 080–8555, Japan; 4 Research Center for Global Agromedicine, Obihiro University of Agriculture and Veterinary Medicine, Obihiro, 080–8555, Japan; 5 Research Center for Advanced Analysis (NAAC), National Agriculture and Food Research Organization (NARO), Tsukuba, 305–8604, Japan; 6 RIKEN Center for Sustainable Resource Science, Yokohama, 230–0045, Japan

**Keywords:** soil bacterial community, rhizobial inoculation, *Bradyrhizobium*, land usage, 16S rRNA amplicon sequence

## Abstract

Although microbial inoculation may be effective for sustainable crop production, detrimental aspects have been argued because of the potential of inoculated microorganisms to behave as invaders and negatively affect the microbial ecosystem. We herein compared the impact of rhizobial inoculation on the soil bacterial community with that of agricultural land-use changes using a 16S rRNA amplicon ana­lysis. Soybean plants were cultivated with and without five types of bradyrhizobial inoculants (*Bradyrhizobium diazoefficiens* or *Bradyrhizobium ottawaense*) in experimental fields of Andosol, and the high nodule occupancy (35–72%) of bradyrhizobial inoculants was confirmed by *nosZ* PCR. However, bradyrhizobial inoculants did not significantly affect Shannon’s diversity index (α-diversity) or shifts (β-diversity) in the bacterial community in the soils. Moreover, the soil bacterial community was significantly affected by land-use types (conventional cropping, organic cropping, and original forest), where β-diversity correlated with soil chemical properties (pH, carbon, and nitrogen contents). Therefore, the effects of bradyrhizobial inoculation on bacterial communities in bulk soil were minor, regardless of high nodule occupancy. We also observed a correlation between the relative abundance of bacterial classes (*Alphaproteobacteria*, *Gammaproteobacteria*, and *Gemmatimonadetes*) and land-use types or soil chemical properties. The impact of microbial inoculation on soil microbial ecosystems has been exami­ned to a limited extent, such as rhizosphere communities and viability. In the present study, we found that bacterial community shifts in soil were more strongly affected by land usage than by rhizobial inoculation. Therefore, the results obtained herein highlight the importance of assessing microbial inoculants in consideration of the entire land management system.

Microbial inoculants in crop cultivation are promising for enhancing plant nutrient uptake and protecting plants against abiotic and biotic stressors (Lau and Lennon, 2012; Petipas *et al.*, 2017). These inoculants are indispensable for sustainable agriculture because of their potential to reduce the use of synthetic fertilizers and pesticides. Their usage is anticipated to increase in the future, and the market for these microbial inoculants is expanding (Waltz, 2017; Chen *et al.*, 2018; Santos *et al.*, 2019).

However, microbial inoculation needs to be judiciously applied due to the potential for an inoculum to become an invader and negatively impact the microbial ecosystem because the behavior of microorganisms in ecosystems is unpredictable (Jack *et al.*, 2021). Although the effects of inoculants, such as rhizobia, mycorrhiza, plant growth-promoting rhizobacteria (PGPR), and biocontrol microbes, on the microbial community in the rhizosphere have been exami­ned (Trabelsi and Mhamdi, 2013; Mawarda *et al.*, 2020), limited information is currently available on their effects on the microbial community in bulk soils (Mayerhofer *et al.*, 2017; Xiong *et al.*, 2017). Furthermore, the effects of microbial inoculation and other anthropogenic interventions have not yet been compared in agricultural settings, where changes in land use (crop cultivation, fertilization, and tillage) may affect the microbial community in soil (Marschner *et al.*, 2003; Ashworth *et al.*, 2017).

Rhizobia are among the most effective bioinoculants because they form root nodules in leguminous plant roots and symbiotically supply fixed nitrogen to the host plants. *Bradyrhizobium* have been used as microbial inoculants for soybean cultivation in many countries from as far back as 130 years ago (Santos *et al.*, 2019). One of the challenges associated with rhizobial material development is that indigenous rhizobia mask the inoculation effect (competition problem) because some indigenous rhizobia have high compatibility in nodulation, but do not contribute to host growth or yield (Triplett and Sadowsky, 1992).

We constructed a mixture of bradyrhizobial strains isolated from various soils with a view to developing a bradyrhizobial inoculum that overcomes the competition problem (Akiyama *et al.*, 2016). Their effectiveness is not limited to symbiotic nitrogen fixation, they also drive the reduction of N_2_O (greenhouse gas) emitted from soybean root systems during the late soybean growth period (Itakura *et al.*, 2013; Akiyama *et al.*, 2016). In our previous studies, N_2_O emission was mitigated at the field scale following an inoculation with *Bradyrhizobium diazoefficiens* carrying the N_2_O reductase gene (*nosZ*) (Itakura *et al.*, 2013; Akiyama *et al.*, 2016). *Bradyrhizobium ottawaense* members have recently been shown to harbor the *nosZ* gene (Wasai-Hara *et al.*, 2020) and its high expression and strong N_2_O reductase activity have been demonstrated (Wasai-Hara *et al.*, 2023).

In the present study, we investigated the impact of bradyrhizobial inoculation on the bacterial community of bulk soil in soybean fields to clarify whether microbial inoculants markedly affect the microbial soil ecosystem as invaders. The microbial community shifts induced by bradyrhizobial inoculants were compared with those induced by general agricultural management practices, such as no-till farming (organic cropping) and agricultural land development in neighboring experimental plots. The criterion for detrimental community shifts caused by bradyrhizobial inoculation did not exceed those caused by these general agricultural management practices.

## Materials and Methods

### Bacterial strains, media, and construction of a cell mixture

The strains used in the present study are listed in Table S1. C110, D110, and X110 are cell mixtures of 63, 25, and 19 strains of *B. diazoefficiens*, respectively, with all strains being isolated from Japanese soil (Shiina *et al.*, 2014; Minamisawa *et al.*, unpublished) and phylogenetically close to *B. diazoefficiens* USDA110. D110 strains were isolated in the present study (see below for details). Regarding *B. ottawaense*, SG09 is a single inoculum of *B. ottawaense* strain SG09, while BWmix is a cell mixture of 11 strains of *B. ottawaense* (Wasai-Hara *et al.*, 2020; Minakata *et al.*, 2023; Wasai-Hara *et al.*, 2023). All these strains carried the N_2_O reductase gene (*nosZ*), and were identified using an *in silico* polymerase chain reaction (PCR) with bradyrhizobial species-specific *nosZ* primers as follows: component strains C110, D110, and X110 were the *B. diazoefficiens* type (BD-type), whereas SG09 and BWmix were the *B. ottawaense* type (BW-type) (Table S1). Details on *nosZ* primers and the genome ana­lysis are described below.

The preparation and inoculation of bacterial cells and soybean cultivation were performed as previously described with some modifications (Akiyama *et al.*, 2016). Briefly, each strain was grown individually at 30°C for 4 days in HM medium (Cole and Elkan, 1973) supplemented with 0.1% L-arabinose (w/v) and 0.025% (w/v) yeast extract under linear shaking. After estimating cell concentrations based on turbidity at OD_660_ (optical density at 660‍ ‍nm, measured using a spectrometer), cultures were mixed in equal cell amounts.

### Study sites

To effectively compare the effects of rhizobial inoculation and land use on soil bacterial communities, experimental fields at the National Agriculture and Food Research Organization (NARO), Japan (36°01′N, 140°07′E) were selected for their uniform soil origin and climate conditions. These included general cultivation fields (Fields A-1 and A-2), a rotation field with summer soybean/winter barley (Fields B-1 and B-2), and two separated secondary forests (Forests A and B).

Between the 1960s and 2018, Fields A-1 and A-2 were managed as a single plot under conventional crop cultivation (*e.g.*, corn, soybeans, and cabbage) using chemical fertilizer and tillage. In 2019, this plot was divided into two separate fields. Fields B-1 and B-2 have been dedicated to the crop rotation system of summer soybean and winter barley since their establishment in 1983 (Wagai *et al.*, 2013). Field B-1 received chemical fertilizers (NPK), liming, and tillage, whereas Field B-2 was treated annually with fallen tree leaf compost and managed without liming or tillage. Forests A and B are secondary forests that have remained undisturbed since at least the 1960s. The main tree species are conifer (*Pinus densiflora*) and evergreen oaks (*Quercus myrsinifolia Blume*). The soil type in all experimental fields was classified as Andosol. The land-use histories and locations of each field are summarized in Table 1 and Fig. S1, respectively.

### Experimental set-up

**Fig. 1.**
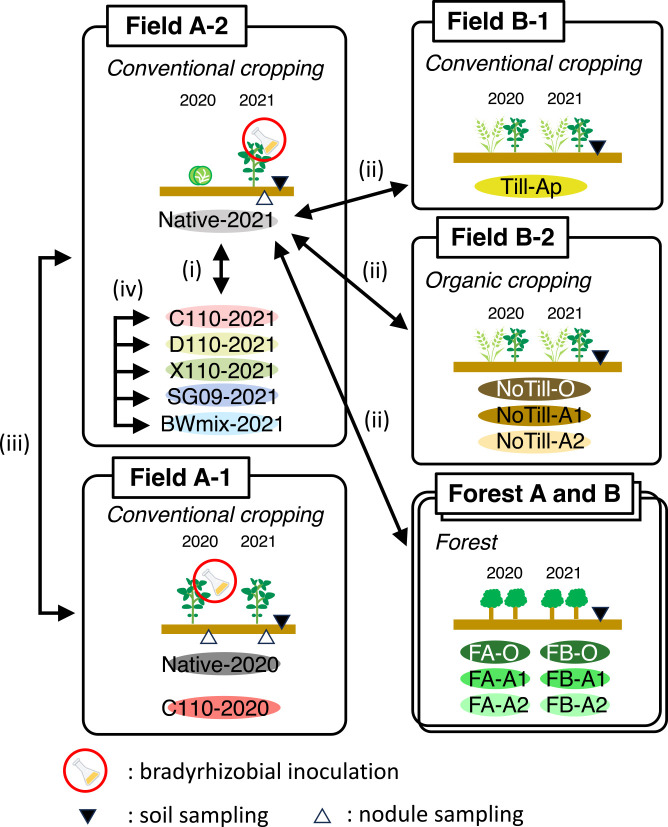
Outline of the experimental design. Numbers near the black arrows indicate factors assessed by comparing the respective soil bacterial communities or nodule occupancy: (i) bradyrhizobial inoculation, (ii) land usage, (iii) time after bradyrhizobial inoculation, (iv) types of inoculants.

The framework of the experimental set-up is shown in Fig. 1. The effects of bradyrhizobial inoculation on the soil bacterial community were evaluated in the conventional cropping plots, Fields A-1 and A-2. Conversely, shifts in the bacterial community due to long-term land-use differences were assessed by comparing the negative control in plot A-2 (“Native-2021”, detailed below) with other plots, including Fields B-1 and B-2 and Forests A and B. Additionally, the effects of short-term land-use changes between 2019 and 2021 were exami­ned by comparing negative controls in Fields A-2 and Field A-1 (“Native-2021” and “Native-2020”, respectively).

Fields A-1 and A-2 were treated with bradyrhizobia by transplanting soybean seedlings inoculated with one of five bradyrhizobial inoculants (C110, D110, X110, SG09, and BWmix) or indigenous bradyrhizobia in field soil (Native). Considering the effects of time after the inoculation and differences in the types of inoculants, bradyrhizobial inoculation trials were conducted as follows. Seedlings inoculated with C110 and Native were transplanted to Fields A-1 and A-2 in 2020 and 2021, respectively. Seedlings inoculated with four different bradyrhizobial inoculants other than C110 were transplanted to Field A-2 in 2021, as were C110 and Native.

In Field A-1, which was not cropped in 2019, soybean seedlings inoculated with C110 or soil from Field A-1 were transplanted into the field in 2020. Following inoculation in 2020, additional inoculation was not conducted in 2021, and soybean seeds were sown directly in the field to monitor the fate of the C110 inoculant in the soil. Each treatment was named “C110-2020” and “Native-2020,” respectively.

In Field A-2, cabbage was cultivated in 2019 and in 2020, and soybean seedlings inoculated with one of the five bradyrhizobial inoculants (C110, D110, X110, SG09, and BWmix) or soil from Field A-2 was transplanted into the field in 2021. The treatments were named “C110-2021”, “D110-2021”, “X110-2021”, “SG09-2021”, “BWmix-2021”, and “Native-2021”, respectively. Before the cultivation, both fields received tillage, liming, and basal fertilizer (30‍ ‍kg N ha^–1^, as ammonium-N, 100‍ ‍kg ha^–1^; P_2_O_5_ equivalent, 100‍ ‍kg ha^–1^; K_2_O equivalent) each year.

### Inoculation procedures and soybean growth

In the inoculation treatment in 2020 (C110-2020 and Native-2020), soybean (*Glycine max* L. Merr., ver. Hatayutaka) seeds were planted in biodegradable Jiffy pots (Jiffy International AS) filled with Andosol soil on June 16, 2020, with a lower population of native soybean bradyrhizobia than in the experimental field, which were collected from an orchard located approximately 100‍ ‍m from the experimental field (Akiyama *et al.*, 2016), and inoculated with 4.0×10^9^ cells of a bradyrhizobial inoculant (C110) in 15‍ ‍mL of peat moss or 0.1‍ ‍g of Field A-1 soil (Native) per pot.

In the inoculation treatment in 2021 (C110-2021, D110-2021, X110-2021, SG09-2021, BWmix-2021, and Native-2021), germinated soybean seeds were planted on June 22, 2021 in biodegradable Jiffy pots filled with Andosol soil collected from an orchard and then inoculated with 4.0×10^9^ cells of one of the bradyrhizobial inoculants (C110, D110, X110, SG09, and BWmix) in 1‍ ‍mL sterilized water or 0.1‍ ‍g of Field A-2 soil (Native) per pot.

Soybean seedlings were grown in a greenhouse under natural light and transplanted with their biodegradable pots into Field A-1 on June 22, 2020 and Field A-2 on June 30, 2021. On August 3, 2020 and August 23, 2021, three plants were randomly selected from each plot. Nodules larger than 1‍ ‍mm in diameter were manually collected from these plants, pooled into one nodule sample for each plot, and stored at –80°C.

### Analysis of nodule occupancy by *nosZ* PCR

Nodule DNA was directly extracted from each nodule as previously described with some modifications (Saeki *et al.*, 2000). In brief, nodules were crushed in 150‍ ‍μL distilled sterile water with a sterile toothpick, 75‍ ‍μL of the crushed solution was transferred to a 96-well plate, and the other 75‍ ‍μL was mixed with 25‍ ‍μL of 50% glycerol and stored at –80°C for bacterial isolation. Crushed solutions in the 96-well plate were centrifuged (4,000‍ ‍rpm, room temperature, 10‍ ‍min), and the supernatant was removed. Fifty microliters of 1% NaCl solution was then added to each well, and the supernatant was removed by centrifugation (4,000‍ ‍rpm, room temperature, 10‍ ‍min). Forty microliters of distilled sterile water was added to each well, and the suspension was transferred to a new 96-well PCR plate. Fifty microliters of BL buffer (40‍ ‍mM Tris, 1% Tween20, 0.5% Nonidet P-40, and 1‍ ‍mM EDTA, pH 8.0) and 10‍ ‍μL proteinase K (1‍ ‍mg mL^–1^) were added to each well, suspended, and incubated at 60°C for 20‍ ‍min and then at 95°C for 5‍ ‍min. After centrifugation (1,500‍ ‍rpm, room temperature, 1‍ ‍min), the supernatant solution was used as the template for PCR.

PCR was performed using *nosZ*-specific primers for *B. diazoefficiens* and *B. ottawaense*. Bradyrhizobial species-specific *nosZ* primers were as follows: nosZ_BD-F/nosZ_BD-R for 832 bp of *B. diazoefficiens nosZ*, and nosZ_BW-F/nosZ_BW-R for 499 bp of *B. ottawaense nosZ* (Table S2). Nodule DNAs were amplified by multiplex PCR using the Blend Taq plus buffer and DNA polymerase system (TOYOBO). A multiplex PCR reaction was performed in a volume of 15‍ ‍μL containing 1.5‍ ‍μL of 10× Brend Taq Buffer, 1.5‍ ‍μL of 2‍ ‍mM each dNTP Mix, 1‍ ‍μL of DNA template, and 0.4‍ ‍μM of each primer. The PCR temperature profile was set at 94°C for 2‍ ‍min, followed by 25 cycles at 94°C for 45‍ ‍s, 56°C for 45‍ ‍s, and 72°C for 45‍ ‍s using the T100 Thermal Cycler (BIO-RAD). PCR products were resolved on a 1% agarose gel, stained with Midori Green Xtra (Nippon Genetics), and visualized using a Blue/Green LED transilluminator. Nodule occupancy (%) was calculated as the ratio of each positive band size to the total number of analyzed nodules.

### Isolation and selection of bradyrhizobial strains for D110

To construct D110, *Bradyrhizobium* strains harboring BD-type nosZ, which were detected using *nosZ*-specific primers for *B. diazoefficiens* (nosZ_BD-F/nosZ_BD-R), were selected from the nodules of field grown soybeans inoculated with C110 (C110-2020). Freeze-stocked nodule solutions of rhizobia with BD-type nosZ in 2020 were streaked onto HM agar plates and 121 strains were isolated. The isolates were subjected to PCR using nosZ_BD-F/nosZ_BD-R for confirmation, and the internal transcribed spacer (ITS) region was identified as *B. diazoefficiens*. Twenty-five representative strains were selected based on the genomic ana­lysis and used as D110.

### Whole-genome sequencing of bradyrhizobial strains

The genomic DNA of bradyrhizobial strains (C110, D110, and F1-1 in BWmix) was extracted from cell cultures in HM medium. Bacterial cells in 10‍ ‍mL of growth medium were collected via centrifugation at 3,500×*g* for 10 min and washed with STE/TNE buffer (pH 8.0). Cell pellets were lysed in 1.2‍ ‍mL of TE buffer containing 0.5‍ ‍mg mL^–1^ of lysozyme and then incubated at room temperature for 10‍ ‍min. A total of 1.5‍ ‍mL of TE buffer containing 0.2‍ ‍mg mL^–1^ pronase and 2.3% SDS was added to the cells, followed by an incubation at 37°C for 2–3 h. The cleared lysate was processed using phenol extraction, chloroform/isoamyl alcohol extraction, ethanol precipitation, and RNA digestion. Genomic DNA was dissolved in TE buffer and kept at –20°C.

Library construction, whole-genome sequencing using the PacBio Sequel II system (Pacific Biosciences), and assembly using Hierarchical Genome Assembly Process v.4 within SMRTlink were performed as previously described (Shimasaki *et al.*, 2021; Nakayasu *et al.*, 2023). The draft genome of strain A2C-5 at D110 was exami­ned as previously described, with some modifications (Hara *et al.*, 2019), *i.e.*, the MiSeq Reagent Kit (Illumina; v3, 2×300 cycles) version and the assembly parameters in CLC Genomics Workbench v. 8.5.1 software (CLC Bio; ambiguous limit, 2; quality limit, 0.05; number of 5′ terminal nucleotides, 20; number of 3′ terminal nucleotides, 10; minimum number of nucleotides in reads, 220) used in the present study were different.

Sequence data were deposited in the DNA Data Bank of Japan (DDBJ) Sequence Read Archive under the accession numbers shown in Table S1.

### Soil sampling

Bulk soils of Field A-1, Field A-2, Field B-1, Field B-2, Forest A, and Forest B, which were of the same soil type (Andosol), but with different land uses (Table 1), were collected for a bacterial community ana­lysis on November 16 and 25, 2021. The shoots of soybean plants had been harvested, while the roots were left behind in Fields A-1, A-2, B-1, and B-2.

In Fields A-1 and A-2, soils up to a depth of 20‍ ‍cm were sampled using a hand shovel at four random locations from the furrow to obtain one composite sample per plot. Plant residues were visually removed from soil samples. Fields B-1 and B-2 were divided into three subplots, and soils to a depth of 20‍ ‍cm were sampled using a soil core at four random locations from the furrows to obtain one composite sample per subplot. In Field B-1, soil samples were collected from the entire plowed horizon (0–20‍ ‍cm, Till-Ap). In Field B-2, soil samples were collected from the organic horizon (NoTill-O), C-rich horizon (0–5‍ ‍cm, NoTill-A1), and illuviated horizon within a previously plowed horizon (5–20‍ ‍cm, NoTill-A2). The samples obtained were passed through a 2-‍mm sieve.

At the forest sites (Forest A and Forest B), three line transects were established, and soils to a depth of 20‍ ‍cm were sampled by coring at four points in the transect to make one composite sample. In Forest A, soil in the organic horizon (FA-O), shallow soil in A horizon (0–5‍ ‍cm, FA-A1), and deeper soil within A horizon (5–20‍ ‍cm, FA-A2) were collected. FB-O, FB-A1, and FB-A2 were collected from Forest B in a similar manner. The samples obtained were passed through a 2-mm sieve.

All soil samples were stored at –80°C until DNA extraction. Soil properties were analyzed using air-dried soil as previously described (Wagai *et al.*, 2013). Briefly, soil pH was measured using a 1:2.5 (w/v) soil/water suspension. Total carbon (TC) and total nitrogen (TN) contents were measured using an elemental analyzer (vario MAX cube, Elementar Analysensysteme GmbH) or CN Analyzer (NC-22F, Sumika Chemical Analysis Service). In carbon and nitrogen measurements of FB-O, we resampled from the same plot on March 27, 2023 because samples remaining from the previous collection were insufficient for ana­lyses. Soil properties are shown in Table S3.

### Soil DNA extraction and MiSeq sequencing

DNA was extracted from soil samples (500‍ ‍mg) using Extrap Soil DNA Kit Plus Ver.2 (BioDynamics Laboratory) according to the manufacturer’s instructions. The 16S and *nosZ* clade I amplicon libraries for MiSeq sequencing were prepared using a two-step PCR method according to the 16S Metagenomic Sequencing Library Preparation protocol (https://support.illumina.com/downloads/16s_metagenomic_sequencing_library_preparation.html). In the first PCR, we used primers specific for 16S V3-V4 (Klindworth *et al.*, 2013) and *nosZ* clade I (Zhang *et al.*, 2021) with Illumina overhang adapter sequences, as summarized in Table S2. To reduce PCR and sequencing errors and evaluate PCR biases, we employed MAUI (metabarcoding using amplicons with unique mole­cular identifier)-seq technology (Fields *et al.*, 2021), in which the forward primer contained 12-base unique mole­cular identifiers (UMIs). The first PCR products were subjected to a second PCR to attach dual indices and Illumina sequencing adaptors using Nextera XT Index Kit v2 (Illumina). Amplicon libraries were subjected to paired-end sequencing on a MiSeq platform using the Illumina MiSeq Regent Kit v.3 (2×300 bp).

### Bioinformatics

Based on the criteria described by Bamba *et al.* (2024), we performed quality control on Illumina raw reads, the assembly of paired-end reads, and counting UMIs. Briefly, trimmed reads with Phred scores of 20 were assembled using PEAR v0.9.6 (Zhang *et al.*, 2014). The abundance of unique sequences generated from the dereplication of these assembled reads was assessed based on the number of UMIs using the Python 3 scripts described by Bamba *et‍ ‍al.* (2024). Sequences with an average number of UMIs <0.1 for all samples were discarded. Sequence data have been deposited in the DDBJ Sequence Read Archive under accession numbers DRR524638–DRR524697 for 16S V3-V4 and DRR524698–DRR524757 for *nosZ* clade I.

To process 16S rRNA UMIs tables, Mothur software (Schloss *et al.*, 2009) and the SILVA database (release 132, Quast *et al.*, 2013) were used for filtering out non-target sequences, clustering into amplicon sequence variants (ASVs), and identifying taxonomies. After ASV table construction, 24,301–40,624 sequences remained per sample. The sequence reads of each sample were rarefied to 24,301 reads per sample, and percent relative abundance, α-diversity, and β-diversity were calculated using the phyloseq package (version 1.44.0, McMurdie and Holmes, 2013) and vegan package (version 2.6-6.1, Oksanen *et al.*, 2024) in R software (version 4.3.1, https://www.r-project.org). To visualize similarities between microbial communities, the Bray-Curtis dissimilarity matrix was calculated and visualized by non-metric multidimensional scaling (NMDS) using the VEGAN package in R. The “envfit” function in VEGAN was applied to fit soil environmental factors with a significance level of *P*<0.001 onto NMDS ordination.

To process *nosZ* clade I UMI tables, Mothur software and a FunGene database (Fish *et al.*, 2013) were used to filter non-target sequences and cluster them into ASV. After ASV table construction, 1,089–11,542 sequences per sample remained. The sequence reads of each sample were rarefied to 1,089 reads per sample, and percent relative abundance was calculated using the phyloseq package.

### Statistical ana­lysis

Statistical ana­lyses of nodule occupancy, the diversity index, and the relative abundance of bacterial taxa were performed using R software. Correlations between the relative abundance of bacterial taxa and soil properties were estimated by calculating Spearman’s ρ values using R software.

## Results

### Nodule occupancy of inoculated bradyrhizobia

To confirm the nodule occupancy of inoculated bradyrhizobia, three individual plants were randomly selected from each plot in Fields A-1 and A-2 in 2020 and 2021, and nodules were collected. The composition of nodule-forming rhizobia in each plot was estimated by *nosZ*-PCR (BD-type, BW-type, or others) for 91 individual nodules per plot following direct DNA extraction from each nodule (Table 2). In Field A-1 in 2020, the BD-type occupancy of the C110-2020 treatment was 43.8%, which was significantly higher than 0.7% occupancy in Native-2020. In 2021, when soybeans were grown without additional inoculation in Field A-1, the occupancy of the BD type in C110-2020 was 35.3%, which was significantly higher than 1.6% occupancy in Native-2020 (Table 2). In 2021, the occupancies of the same type as the inoculant in C110-2021, D110-2021, X110-2021, SG09-2021, and BWmix-2021 were 61.0, 72.3, 70.3, 57.1, and 43.7%, respectively, which were significantly higher than those in Native-2021.

Increased nodule occupancy by the same *nosZ* type bradyrhizobia used as the inoculant in Fields A-1 and A-2 confirmed effective bradyrhizobial inoculation in terms of nodule formation (Table 2). Notably, in Field A-1 in 2021, where soybeans were grown without additional inoculation, BD-type occupancy was significantly higher in C110-2020 than in Native-2020 (Table 2).

### Relative abundance of ASVs identical to bradyrhizobial inoculants in bulk soil

**Fig. 2.**
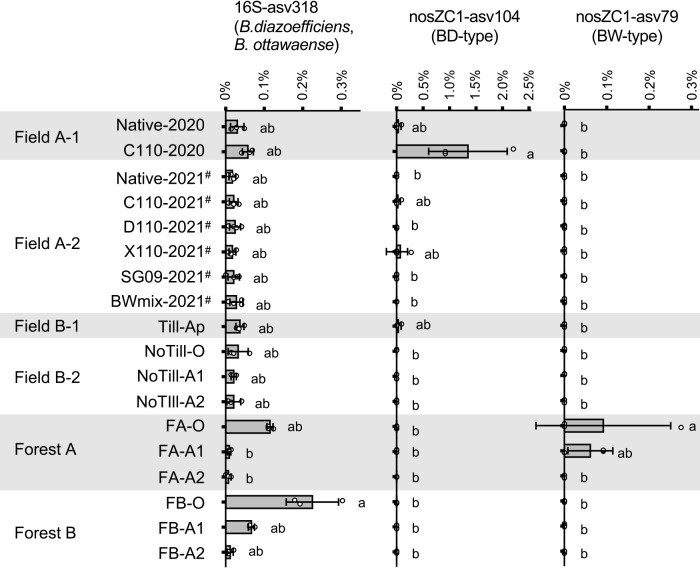
Relative abundance of ASVs (16S rRNA and nosZ clade I) identical to bradyrhizobial inoculums. The sequence of 16S-asv318 was identical to those of inoculants and reference strains of *Bradyrhizobium diazoefficiens* and *Bradyrhizobium ottawaense*. The sequences of nosZC1-asv104 and nosZC1-asv79 were identical to BD-type inoculants (C110, D110, and X110) and BW-type inoculants (SG09 and BWmix), respectively. Different letters indicate a significant difference (*P*<0.05, the Steel-Dwass test). Values are means±SD (*n*=3 or 4^#^).

The 16S rRNA sequences of inoculated bradyrhizobial strains in the present study were identical to that of 16S-asv318, which was identical to those of *B. diazoefficiens* USDA110^T^ and *B. ottawaense* OO99^T^ (Fig. S2A). The relative abundance of 16S-asv318 in Fields A-1 and A-2 was 0.02–0.06% regardless of bradyrhizobial inoculation and was higher in the O horizon of Forests A and B at 0.11 and 0.22%, respectively (Fig. 2).

The *nosZ* sequences of the inoculated bradyrhizobial strains (Table S1) were identical to those of nosZC1-asv104 and nosZC1-asv79 (Fig. S2B). The relative abundance of nosZC1-asv104 was the highest in C110-2020 and was almost undetectable in other agricultural and forest soils (Fig. 2). The relative abundance of nosZC1-asv79 was the highest in FA-O, whereas it was not detected in sites other than Forest A.

### Effects of land-use differences and bradyrhizobial inocu­lation on bacterial species richness in bulk soil

**Fig. 3.**
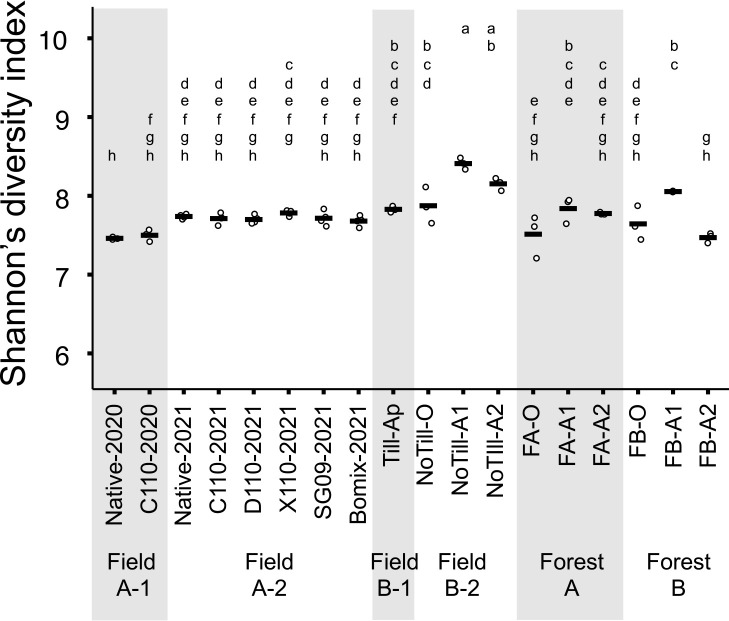
Shannon’s diversity index of soil bacteria. Dots shows raw data and bars represent the mean. Different letters indicate a significant difference (*P*<0.05, Tukey’s HSD test).

The effects of land usage and bacterial inoculants on the species richness of soil bacteria were shown by the Shannon index calculated from the ASV of 16S rRNA (Fig. 3). In Fields A-1 and A-2, no significant differences were observed between the diversity indexes of any inoculation treatment and the Native-2021 treatment. In Fields B-1 and B-2, the diversity indexes of Till-Ap and NoTill-O did not significantly differ from those of Native-2021, while NoTill-A1 and NoTill-A2 had significantly higher diversity indexes than Native-2021 soils. In Forests A and B, diversity indexes did not significantly differ from those of Native-2021, while FB-A1 soil showed a higher diversity index than Native-2021.

### Effects of land-use differences and bradyrhizobial inocu­lation on the bacterial community structure in bulk soil

**Fig. 4.**
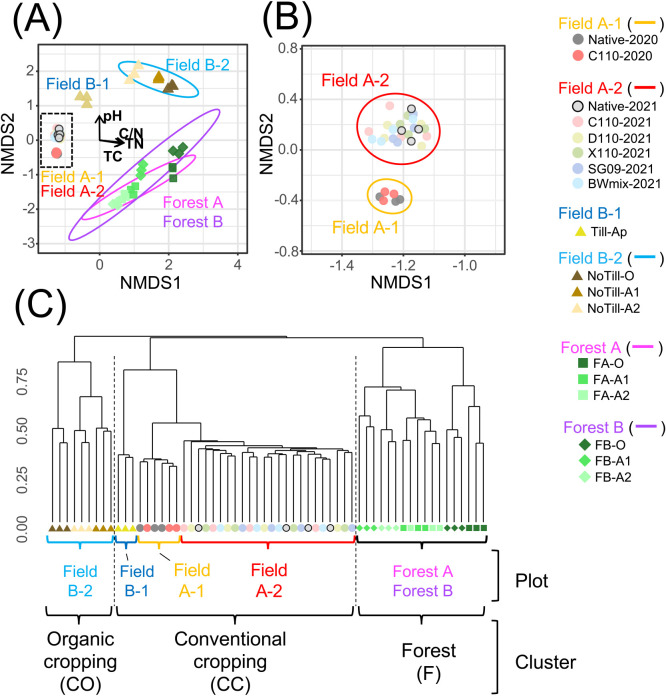
Soil bacterial community structure in soil. (A) Non-parametric multidimensional scaling (NMDS) based on Bray Curtis similarity (stress=0.077). Ellipse lines drawn using the stat_ellipse function in the ggplot2 R package are based on the variance observed between each plot, except for plot B-1, where the sample size was too small. Arrows represent vectors of environmental factors fit with the ordination of the bacterial community using the envfit function in the vegan R package. Length is proportional to the strength of the correlation. Only factors with *P*<0.001 are shown. The direction of the increasing abundance of bacterial taxa is shown in Fig. S3. (B) Enlarged view of the black dashed square in Fig. 4A. (C) Hierarchical cluster ana­lysis using Bray Curtis similarity and Ward’s a clustering algorithm. Clusters correspond to the management type (Table 1).

In non-parametric multidimensional scaling (NMDS) with Bray–Curtis dissimilarity, the bacterial communities of Fields A-1 and A-2 were clearly distinct from those of Fields B-1 and B-2 and Forest AB (Fig. 4A), while the bacterial communities of Fields A-1 (C110-2020 and Native-2020) and A-2 (C110-2021, D110-2021, X110-2021, SG09-2021, BWmix-2021, and Native-2021) were separated (Fig. 4B). In a correlation ana­lysis of differences in community structures and soil properties, the parameters that correlated with the ordination of the entire community were TC, TN, the carbon-to-nitrogen ratio (C/N), and pH (*P*<0.01) (Fig. 4A). Community structure differences in Field A (Field A-1 and A-2) and Forest AB correlated with TC, TN, and C/N. In addition, differences between Field B-2 and Forest AB correlated with differences in pH, and differences between O and the other horizons in Field B-2 and Forest AB correlated with differences in TC, TN, and C/N. In a comparison of the bacterial community and relative abundance gradients of taxa at the class level, a correlation was detected for *Gemmatimonadetes* in Field A-2 (Fig.‍ ‍S3). Furthermore, correlations were observed for *Alphaproteobacteria* and *Gammaproteobacteria* in Forest AB (Fig. S3). In Field B-2 and Forest AB soils, community shifts were consistent with soil depths (Fig. 4A).

**Fig. 5.**
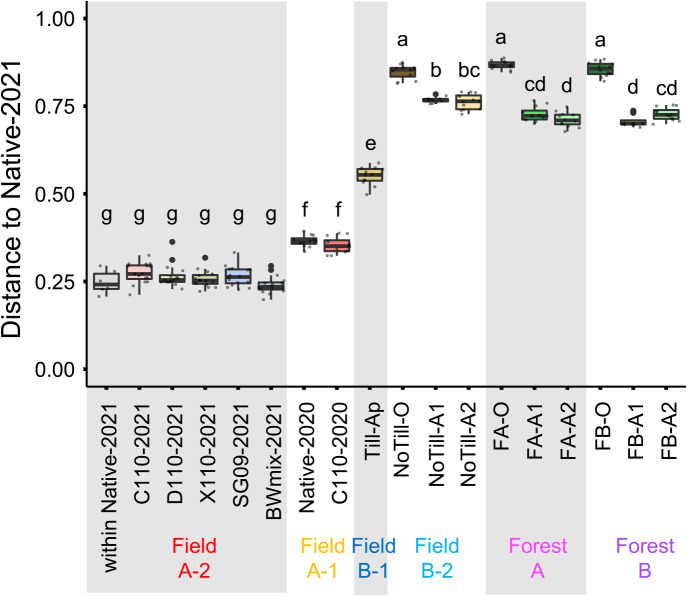
Distance to Native-2021 in non-parametric multidimensional scaling (NMDS) using Bray Curtis similarity. Box plots show means and variance in distances to Native-2021 in Fig. 4A. Different letters indicate a significant difference (*P*<0.05, the Steel-Dwass test).

A comparison of distances from Native-2021 in NMDS plots showed that Field A-1, Field B-1, Field B-2, and Forest AB plots were significantly different from Field A-2 plots (Native-2021, C110-2021, D110-2021, X110-2021, SG09-2021, and BWmix-2021), while there were no significant differences in Field A-2 soils, including those within Native-2021 (Fig. 5). The distance from Native-2021 to Field B-2 and Forest AB soils was large, while the distance to Field B-1 and Field A-1 soils was smaller, in that order. Native-2020 and C110-2020 were not significantly different in terms of their distances from Native-2021.

### Soil bacterial community under different land management types

In hierarchical clustering results showing the Bray–Curtis distance (Fig. 4C), Field A-1, Field A-2, and Field B-1 soils were classified into the same cluster, while Field B-2 and Forest AB soils formed separate clusters. These clusters were defined as the “Conventional cropping cluster (CC-cluster)”, “Organic cropping cluster (OC-cluster)”, and “Forest cluster (F-cluster)”, corresponding to land management types (Table 1).

**Fig. 6.**
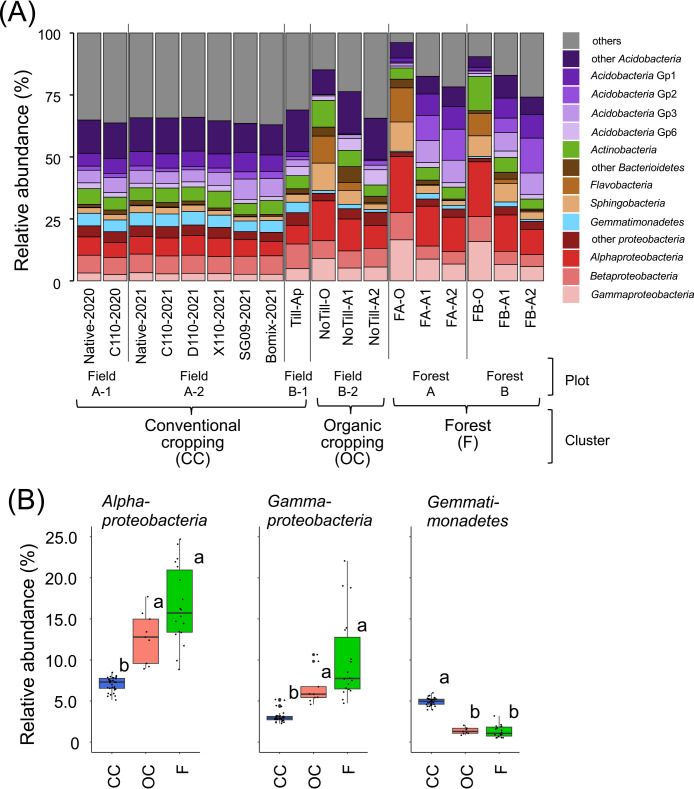
Bacterial communities in bulk soil under different land management types. (A) The bacterial class with relative abundance >5% in at least one treatment is shown with the mean of each treatment. Clusters correspond to the management type (Table 1). (B) Box plots show the relative abundance of *Alphaproteobacteria*, *Gammaproteobacteria*, and *Gemmatimonadetes* among soil management types. Different letters indicate significant differences (*P*<0.05, the Steel-Dwass test). See data on all taxa in Table S4.

The bacterial construction of each cluster is shown at the class level (Fig. 6A and Table S4). The relative abundance of *Alphaproteobacteria* and *Gammaproteobacteria* was significantly lower in the CC-cluster than in the other clusters, whereas that of *Gemmatimonadetes* was significantly higher (Fig. 6B).

### Correlation between the relative abundance of bacterial taxa and soil properties

**Fig. 7.**
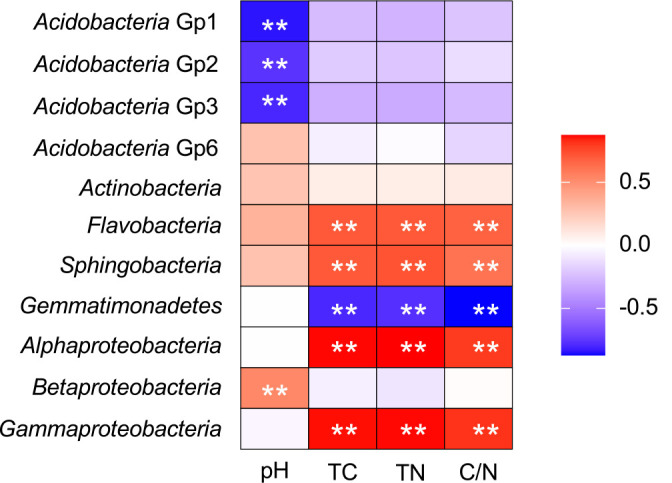
Heat map showing Spearman’s correlation coefficient between the relative abundance of dominant bacterial classes and soil chemical properties. The color scale indicates the correlation coefficient (r). Asterisks denote the correlation that remained after Bonferroni adjustments (**, *P*<0.01). TC, total carbon; TN, total nitrogen; C/N, carbon-to-nitrogen ratio. Scatter plots with a correlation are shown in Fig. S5.

The relative abundance of *Flavobacteria*, *Sphingobacteria*, *Alphaproteobacteria*, and *Gammaproteobacteria* positively correlated with TC, CN, and C/N, whereas that of *Gemmatimonadetes* negatively correlated with these soil properties (Fig. 7 and S4). The relative abundance of *Betaproteobacteria* positively correlated with pH, whereas that of *Acidobacteria* Gp1, *Acidobacteria* Gp2, and *Acidobacteria* Gp3 negatively correlated with pH.

### Relative abundance of bacterial taxa at different soil depths

In soil samples from Field B-2 and Forest AB, the relative abundance of bacterial taxa at different soil depths are shown in Fig. S5. The relative abundance of *Acidobacteria* Gp1, *Acidobacteria* Gp2, and *Acidobacteria* Gp3 was the lowest in the organic horizon and increased with soil depth. In contrast, the relative abundance of *Flavobacteria*, *Sphingobacteria*, and *Alphaproteobacteria* was the highest in the organic horizon and decreased with soil depth. The relative abundance of *Gemmatimonadetes* mostly remained stable regardless of soil depth, but was the highest in A1 horizon soil.

## Discussion

The present study aimed to compare the effects of brady­rhizobial inoculation on soil bacterial community shifts with those of other agricultural management strategies. Prior to the community ana­lysis of soil bacteria, we developed species-specific *nosZ* primers for the detection of inoculants, and the effectiveness of bradyrhizobial inoculation on soybean nodulation was confirmed by a nodule ana­lysis. This method may be useful in trials aimed at inoculating *B. diazoefficiens* or *B. ottawaense* strains in fields or pots dominated by indigenous rhizobia without *nosZ*, such as *B. japonicum* and *B. elkanii*, as in the present study. Rhizobial cells in soybean root nodules are released from senescing nodules into the soil as free-living cells; the nodule type of the soybean root is referred to as the “determinate nodule” (Kondorosi *et al.*, 2013). Therefore, the following scenarios are suggested: inoculated C110 formed nodules in the year of inoculation, was released from the nodule after soybean cultivation, survived in the field soil, and infected soybean plants again next year. The survival of bradyrhizobial inoculants in C110-2020 was supported by the relative abundance of nosZC1-asv104 (Fig. 2). *Bradyrhizobium* are ubiquitous in the soil environment and survive as free-living cells without host plants (Delgado-Baquerizo *et al.*, 2018). In one case, inoculated bradyrhizobial strains were detected in a field 20 years after their introduction without the cultivation of soybean plants for 17 years (Narożna *et al.*, 2015).

We suggested the smaller impact of bradyrhizobial inoculation on the soil bacterial community than other agricultural management practices, particularly by analyzing 16SrRNA amplicon sequences. In the β-diversity ana­lysis, bradyrhizobial inoculation exerted the weakest effects among agriculture-related land management practices considered in‍ ‍this study. Additionally, in the α-diversity ana­lysis, only NoTill-A1, NoTill-A2, and FB-A1 differed from Native-2021, which indicated that the effects of bradyrhizobial inoculation on species richness were similar to or weaker than those‍ ‍of other land-use differences. Furthermore, in the ASV level ana­lysis, the relative abundance of 16S-asv318, which was identical to that of all bradyrhizobial inoculants, was not affected by bradyrhizobial inoculation in Field A-1‍ ‍or A-2. The higher abundance of 16S-asv318 in the O‍ ‍horizon of Forests AB may be due to non-symbiotic *Bradyrhizobium*, which is similar to the findings obtained in‍ ‍a North American forest soil study (VanInsberghe *et al.*, 2015). The reason that *nosZ* ASVs identical to *bradyrhizobial* inoculants were rarely detected in soils from inoculated plots in 2021 may be‍ ‍due to the sampling method; bulk soil was collected from‍ ‍between ridges to avoid nodule contamination. In C110-2020, the soybean stump with nodules was mechanically agitated and spread throughout the soil until the spring of 2021. Therefore, the inoculated bradyrhizobia were expected to remain around the roots until harvesting and then spread throughout the soil by mechanical agitation.

Hierarchical clustering based on β-diversity suggested bacterial community differences in three clusters (CC-, CO-, and F-clusters) that were consistent with land management characteristics. Furthermore, the results obtained showed that the relative abundance of *Gemmatimonadetes*, *Alphaproteobacteria*, *Gammaproteobacteria*, *Flavobacteria*, and *Sphingobacteria* was higher or lower in the CC-cluster than in the two other clusters, which indicated that differences in chemical fertilization, tillage, and liming (the CC cluster vs. OC and F clusters) were factors that more strongly affected relative abundance than differences between agricultural land use and forests (CC- and OC-clusters vs. the F-cluster).

The relative abundance of *Gemmatimonadetes* increased in lower TC, TN, and C/N environments. In contrast, *Flavobacteria*, *Sphingobacteria*, *Alphaproteobacteria*, and *Gammaproteobacteria* positively correlated with TC, TN, and C/N, whereas *Betaproteobacteria* and *Acidobacteria* Gp1, *Acidobacteria* Gp2, and *Acidobacteria* Gp3 correlated with changes in pH. Therefore, the contribution of these soil properties to changes in bacterial communities due to differences in land use is suggested. *Gemmatimonadetes* is the eighth most common taxa in soil (Janssen, 2006), but less than 10 strains have been isolated, and their ecology the environment is still unknown (Mujakić *et al.*, 2022). In studies involving bacterial community ana­lyses of agricultural soils, negative correlations were observed between the relative abundance of *Gemmatimonadetes* and soil organic carbon or TN (Singh *et al.*, 2023), whereas positive correlations were reported in other studies (Wang *et al.*, 2021). Therefore, other soil environmental factors, such as tillage, chemical fertilization, and liming, characteristics of the CC-cluster, may be directly or indirectly related to the growth of *Gemmatimonadetes* in the soil environment.

Bacterial community shifts with soil depth have been observed in forest and non-tilled agricultural field soils (Sun *et al.*, 2018; Mundra *et al.*, 2021). In a long-term continuous no-tillage field in China (Sun *et al.*, 2018), the relative abundance of *Alphaproteobacteria* decreased with soil depth in the 0–20‍ ‍cm range, whereas the relative abundance of *Acidobacteria* increased, which is consistent with the present results. The relative abundance of *Gemmatimonadetes* increased with soil depth (Sun *et al.*, 2018), which differed from the results obtained herein. In Norwegian forests, the relative abundance of *Proteobacteria* decreased and that of *Acidobacteria* increased from the organic horizon to deeper soils in the 0–20‍ ‍cm range (Mundra *et al.*, 2021), which is in agreement with the present results. These common shifts between forest soils and no-till soils may not only be due to the physicochemical gradients associated with soil depth (Fig. S4), but also to direct and indirect relationships with fungi and microeukaryotes that vary with soil depth (Mundra *et al.*, 2021).

In conclusion, the present study demonstrated that brady­rhizobial inoculation exerted weaker effects on the soil bacterial community than other land management practices, and the bacterial community shift was attributed to soil chemical properties. To the best of our knowledge, this is the first study to compare the impact of microbial inoculation on bacterial communities in bulk soil with those of other agricultural activities. To assess the effects of bradyrhizobial inoculation on the microbial community in bulk soil, the fungal community of bulk soil needs to be exami­ned in the future because rhizobial inoculation affects the fungal community in the rhizosphere (Xu *et al.*, 2019). In addition, it is important to monitor changes in the microbial community under repeated rhizobial inoculations, such as the annual inoculation recommended in South American countries (Santos *et al.*, 2019).

## Citation

Hara, S., Kakizaki, K., Bamba, M., Itakura, M., Sugawara, M., Suzuki, A., et al. (2024) Does Rhizobial Inoculation Change the Microbial Community in Field Soils? A‍ ‍Comparison with Agricultural Land-use Changes. *Microbes Environ ***39**: ME24006.

https://doi.org/10.1264/jsme2.ME24006

## Supplementary Material

Supplementary Material 1

Supplementary Material 2

Supplementary Material 3

Supplementary Material 4

Supplementary Material 5

Supplementary Material 6

## Figures and Tables

**Table 1.** Land use history and management type of experimental plots used in the present study

Plot	Sample name	Management type*^a^*	History of land use*^b^*				
			1960s~1982	1983~2018	2019	2020	2021
Field A-1	Native-2020, C110-2020	Conventional cropping	general cropping	general cropping	non-cropping	soybean (I, N)	soybean (D, N, S)
Field A-2	Native-2021, C110-2021, D110-2021, X110-2021, SG09-2021, BWmix-2021	Conventional cropping	general cropping	general cropping	cabbage	cabbage	soybean (I, N, S)
Field B-1	Till-Ap	Conventional cropping	general cropping	soybean/barley	soybean/barley	soybean/barley	soybean/barley (S)
Field B-2	NoTill-O, -A1, -A2	Organic cropping	general cropping	soybean/barley	soybean/barley	soybean/barley	soybean/barley (S)
Forest A	FA-O, -A1, -A2	Forest	secondary forest	secondary forest	secondary forest	secondary forest	secondary forest (S)
Forest B	FB-O, -A1, -A2	Forest	secondary forest	secondary forest	secondary forest	secondary forest	secondary forest (S)

*^a^* Conventional cropping, chemical fertilizers, and tillage; Organic cropping, leaf compost, and no tillage; Forest, untouched secondary forest. *^b^* Letters in parentheses indicate process abbreviations: I, bradyrhizobial inoculation in seedling pots; N, nodule ana­lysis; D, direct sowing in the field without inoculation; S, soil sampling.

**Table 2.** Nodule occupancy in Field A-1 and Field A-2

Plot	Sampling date	Treatment	*nosZ* type of inoculum	Nodule occupancy by *nosZ* PCR*^ab^*			
				BD-type (%)		BW-type (%)	
Field A-1	2020 August 3	Native_2020	—	0.7±1.3	c	NA	
		C110_2020	BD-type	43.8±7.2	b	NA	
	2021 August 23	Native_2020	—	1.6±2.0	c	0±0	c
		C110_2020	BD-type	35.3±4.8	b	0±0	c
Field A-2	2021 August 23	Native_2021^#^	—	1.6±1.4	c	0±0	c
		C110_2021^#^	BD-type	61.0±9.0	a	0±0	c
		D110_2021^#^	BD-type	72.3±8.2	a	0.3±0.5	c
		X110_2021^#^	BD-type	70.3±5.8	a	0.3±0.5	c
		SG09_2021^#^	BW-type	0.3±0.5	c	57.1±11.9	a
		BWmix_2021^#^	BW-type	3.3±5.2	c	43.7±6.4	b

*^a^* Values are shown as means±SD (*n*=3 or 4^#^); NA, not analyzed. *^b^* Different letters indicate significant differences among treatments as assessed using Tukey’s HSD test (*P*<0.05). Gray indicates nodule occupancy (%) corresponding to the *nosZ* types of inoculated bradyrhizobia (BD-type [*Bradyrhizobium diazoefficiens*] or BW-type [*Bradyrhizobium ottawaense*]).
