# Supplementary material for: Does Rhizobial Inoculation Change the Microbial Community in Field Soils? A‍ ‍Comparison with Agricultural Land-use Changes
Source: Microbes Environ. 2024 Sep 12;39(3):ME24006. doi: 10.1264/jsme2.ME24006 (PMC11427313; doi:10.1264/jsme2.ME24006)
Supplement: Supplementary file 1 — Supplementary Material 1 [file 39_24006_s1.pdf]

Fig. S1

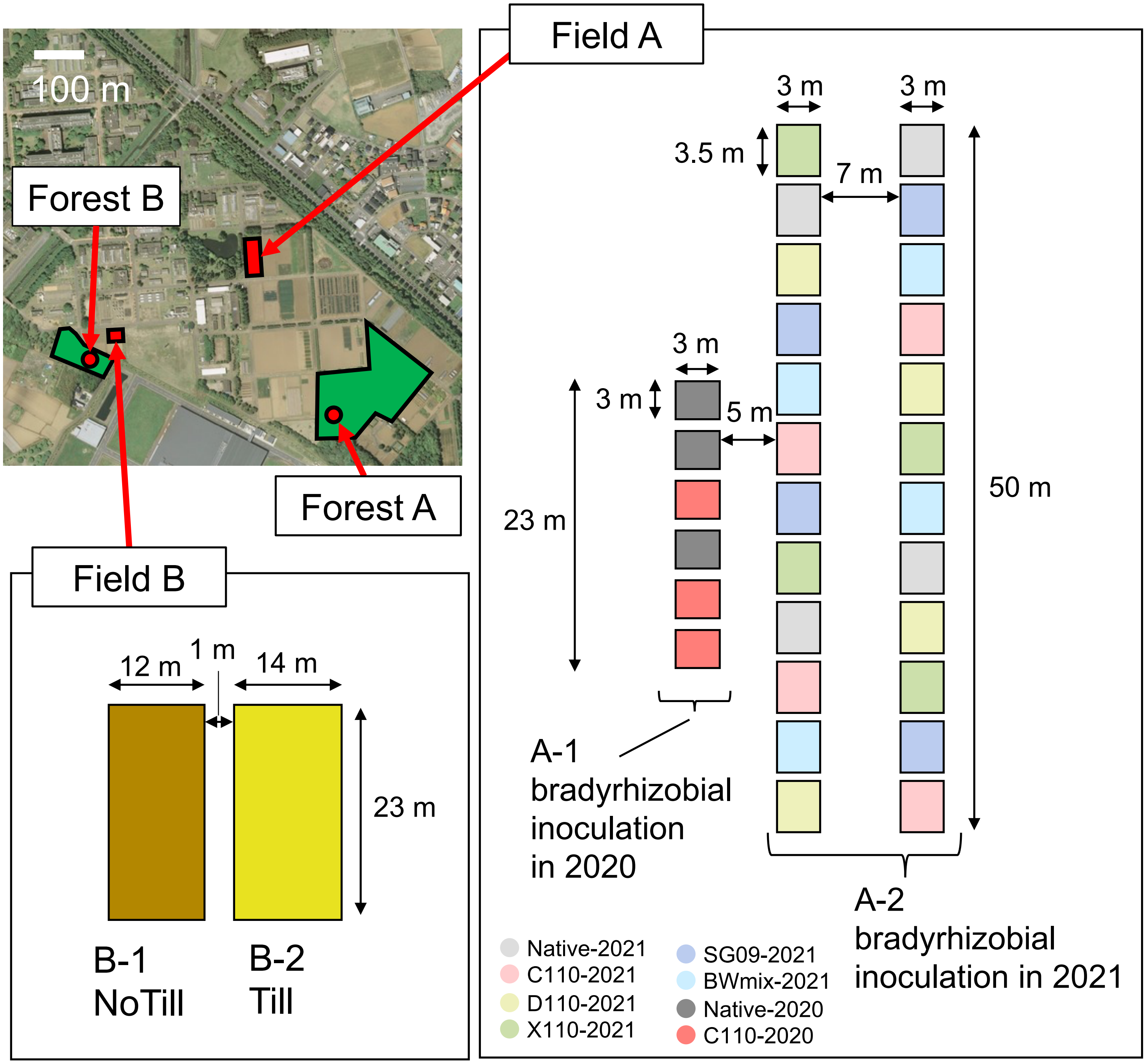

Fig. S1 Map and location of study site.

Source of the aerial photographs: Geospatial Information Authority of Japan (2008)
