# Supplementary material for: Does Rhizobial Inoculation Change the Microbial Community in Field Soils? A‍ ‍Comparison with Agricultural Land-use Changes
Source: Microbes Environ. 2024 Sep 12;39(3):ME24006. doi: 10.1264/jsme2.ME24006 (PMC11427313; doi:10.1264/jsme2.ME24006)
Supplement: Supplementary file 2 — Supplementary Material 2 [file 39_24006_s2.pdf]

Fig. S2

(A) 16S rRNA

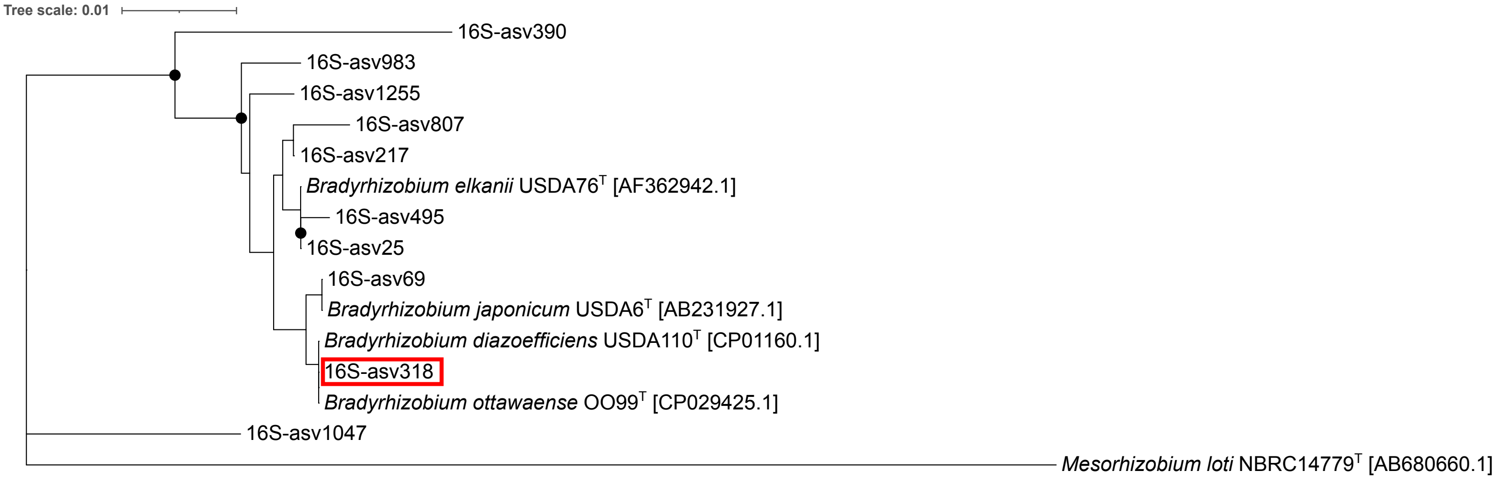

(B) *nosZ* Clade I

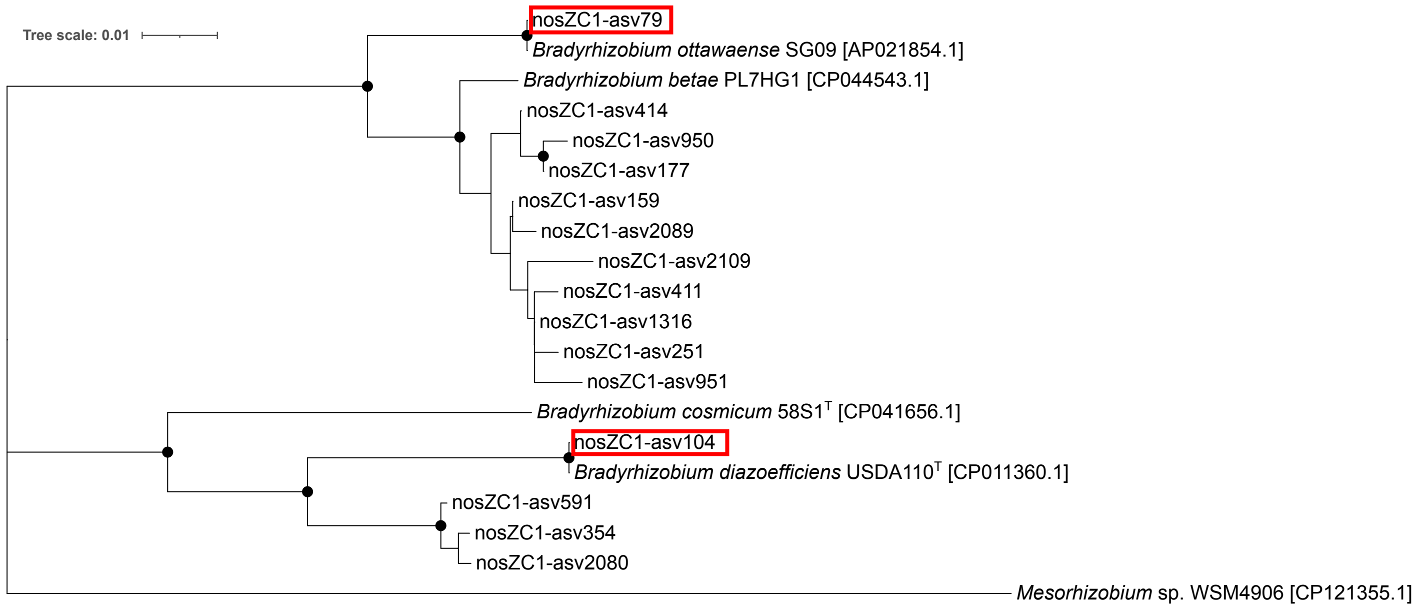

Fig. S2 Phylogenetic tree of (A)16S rRNA genes and (B) nosZ Clade I gene of amplicon sequence variants (ASVs) closed to inoculated bradyrhizobial inoculants constructed by the neighbor-joining method. Red boxes indicate ASVs identical to each sequences of inoculated strains. 16S-asv318 were identical to all strains in C110, D110, X110, SG09, and BWmix. nosZC1-asv104 was identical to strains in C110, D110, and X110, while nosZC1-asv79 was identical to strains in SG09 and BWmix. Branch points supported with bootstrap values of >75% are marked with solid circles.
