# Supplementary material for: Does Rhizobial Inoculation Change the Microbial Community in Field Soils? A‍ ‍Comparison with Agricultural Land-use Changes
Source: Microbes Environ. 2024 Sep 12;39(3):ME24006. doi: 10.1264/jsme2.ME24006 (PMC11427313; doi:10.1264/jsme2.ME24006)
Supplement: Supplementary file 3 — Supplementary Material 3 [file 39_24006_s3.pdf]

Fig. S3

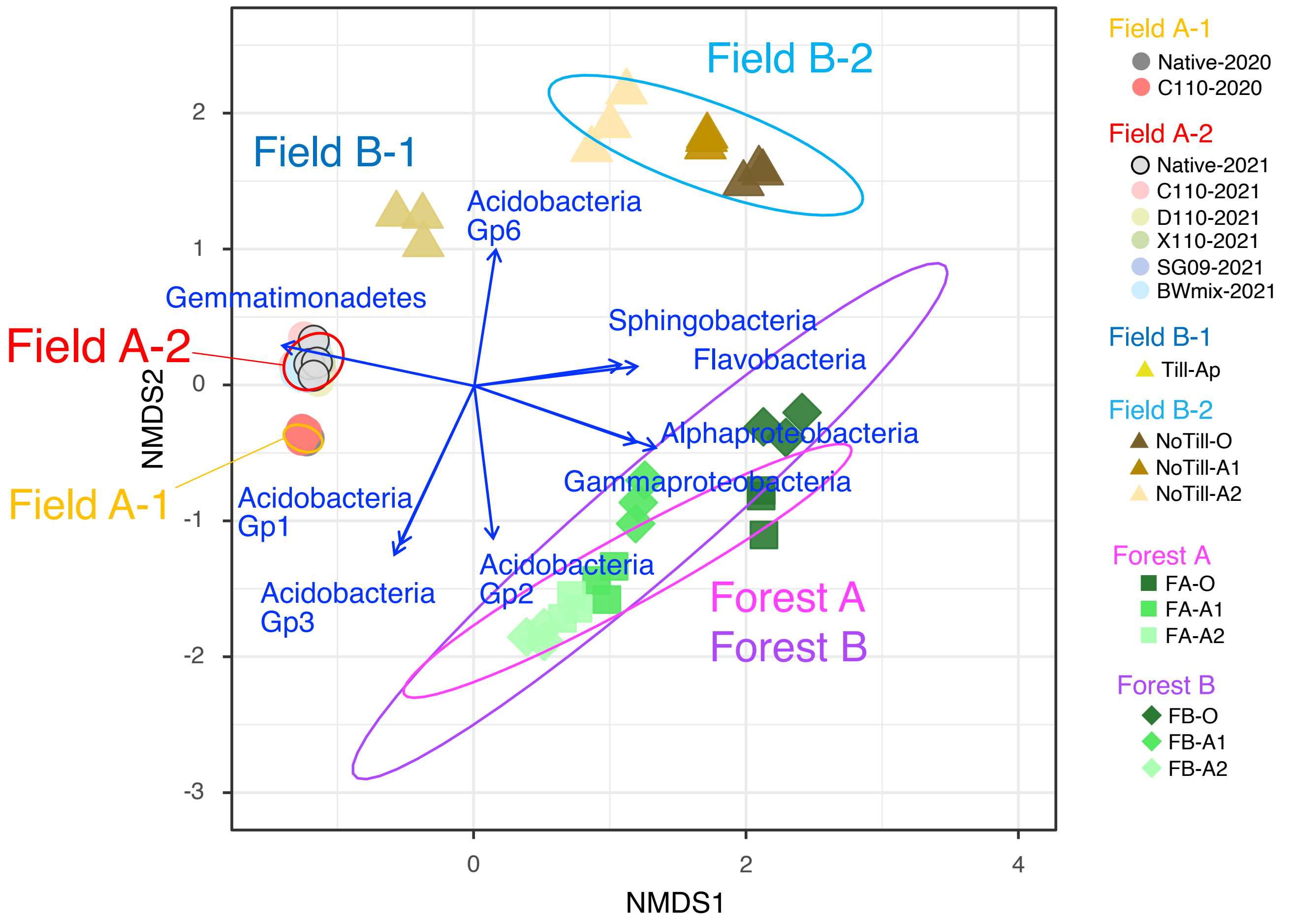

Fig. S3 Nonparametric multidimensional scaling (NMDS) based on Bray-Curtis similarity of bacterial community with direction of increasing abundance of bacterial taxa. Blue arrows represent fitted vectors of continuous associated abundance of each taxa at class level and show the direction of the increasing gradient. Length is proportional to the strength of correlation. Only factors with $p < 0.001$ were shown.
