# Supplementary figures and images for: Does Rhizobial Inoculation Change the Microbial Community in Field Soils? A‍ ‍Comparison with Agricultural Land-use Changes
Source: Microbes Environ. 2024 Sep 12;39(3):ME24006. doi: 10.1264/jsme2.ME24006 (PMC11427313; doi:10.1264/jsme2.ME24006)

Fig. S4

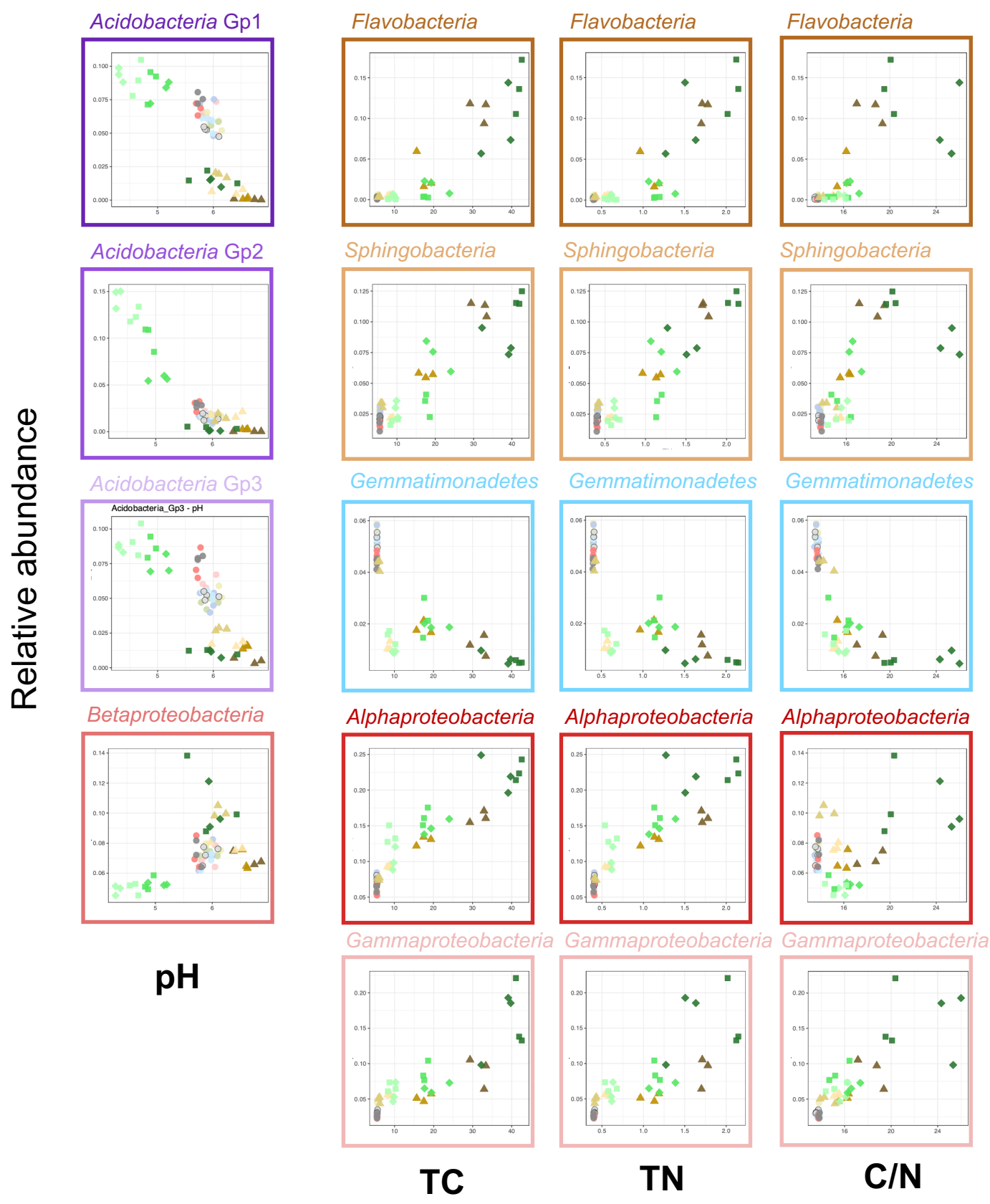

Supplement: Supplementary file 4 — Supplementary Material 4 [file 39_24006_s4.pdf]
