# Supplementary material for: Does Rhizobial Inoculation Change the Microbial Community in Field Soils? A‍ ‍Comparison with Agricultural Land-use Changes
Source: Microbes Environ. 2024 Sep 12;39(3):ME24006. doi: 10.1264/jsme2.ME24006 (PMC11427313; doi:10.1264/jsme2.ME24006)
Supplement: Supplementary file 5 — Supplementary Material 5 [file 39_24006_s5.pdf]

Fig. S5

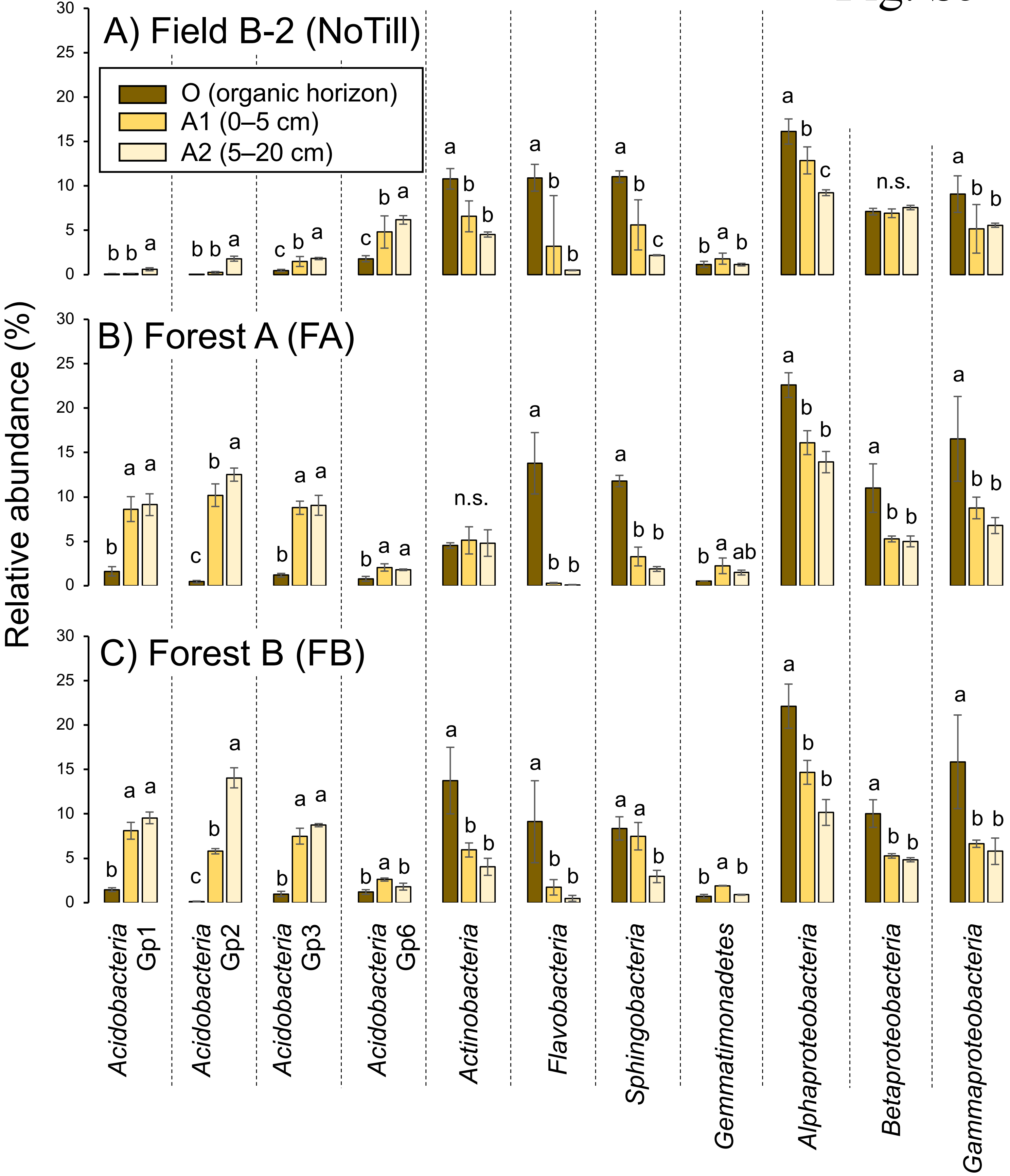

Fig. S5 Barplots displaying abundances distribution of bacterial class in different soil depths. Plots for relative abundances of Field B-2 (A), Forest A (B), and Forest B (C) with soil depth (organic layer (O) and two mineral soil layers: 0–5 cm (A1), 5–20 cm (A2) are shown. Different letters indicate a significant difference among soil layer in same plot ($p < 0.05$, Tukey's test.)
