# Supplementary material for: Does Rhizobial Inoculation Change the Microbial Community in Field Soils? A‍ ‍Comparison with Agricultural Land-use Changes
Source: Microbes Environ. 2024 Sep 12;39(3):ME24006. doi: 10.1264/jsme2.ME24006 (PMC11427313; doi:10.1264/jsme2.ME24006)
Supplement: Supplementary file 6 — Supplementary Material 6 [file 39_24006_s6.pdf]

Table S1 Construction of bradyrhizobal bioinoculants

Strains	Taxonomy	nosZ type[a]	Accession number[b]	Reference
C110				
KS1-16	*B. diazoefficiense*	BD	AP029084	Shiina *et al*., 2014
KS2-2	*B. diazoefficiense*	BD	AP029085	Shiina *et al*., 2014
KS2-3	*B. diazoefficiense*	BD	AP029086	Shiina *et al*., 2014
NG1	*B. diazoefficiense*	BD	AP029087	Shiina *et al*., 2014
NG2	*B. diazoefficiense*	BD	AP029088	Shiina *et al*., 2014
NG5	*B. diazoefficiense*	BD	AP029089	Shiina *et al*., 2014
TS1-1	*B. diazoefficiense*	BD	AP029090	Shiina *et al*., 2014
TS1-2	*B. diazoefficiense*	BD	AP029091	Shiina *et al*., 2014
TS1-3	*B. diazoefficiense*	BD	AP029092	Shiina *et al*., 2014
TS1-4	*B. diazoefficiense*	BD	AP029093	Shiina *et al*., 2014
TS1-5	*B. diazoefficiense*	BD	AP029094	Shiina *et al*., 2014
TS1-6	*B. diazoefficiense*	BD	AP029095	Shiina *et al*., 2014
TS2-1	*B. diazoefficiense*	BD	AP029096	Shiina *et al*., 2014
TS2-10	*B. diazoefficiense*	BD	AP029097	Shiina *et al*., 2014
TS3-4	*B. diazoefficiense*	BD	AP029098	Shiina *et al*., 2014
TS3-5	*B. diazoefficiense*	BD	AP029099	Shiina *et al*., 2014
TS3-6	*B. diazoefficiense*	BD	AP029100	Shiina *et al*., 2014
TS3-7	*B. diazoefficiense*	BD	AP029101	Shiina *et al*., 2014
TS3-19	*B. diazoefficiense*	BD	AP029102	Shiina *et al*., 2014
TS4-125	*B. diazoefficiense*	BD	AP029103	Shiina *et al*., 2014
KM1-1	*B. diazoefficiense*	BD	AP029104 (AP029105)	Shiina *et al*., 2014
KM1-2	*B. diazoefficiense*	BD	AP029106	Shiina *et al*., 2014
KM1-3	*B. diazoefficiense*	BD	AP029107	Shiina *et al*., 2014
KM1-5	*B. diazoefficiense*	BD	AP029108	Shiina *et al*., 2014
KM1-6	*B. diazoefficiense*	BD	AP029109	Shiina *et al*., 2014
KM2-5	*B. diazoefficiense*	BD	AP029110	Shiina *et al*., 2014
KM2-6	*B. diazoefficiense*	BD	AP029111	Shiina *et al*., 2014
KM2-7	*B. diazoefficiense*	BD	AP029112	Shiina *et al*., 2014
KM2-8	*B. diazoefficiense*	BD	AP029113	Shiina *et al*., 2014
YM1-1	*B. diazoefficiense*	BD	AP029114	Shiina *et al*., 2014
YM1-3	*B. diazoefficiense*	BD	AP029115	Shiina *et al*., 2014
YM1-4	*B. diazoefficiense*	BD	AP029116 (AP029117, AP029118)	Shiina *et al*., 2014
YM1-5	*B. diazoefficiense*	BD	AP029119	Shiina *et al*., 2014
YM1-6	*B. diazoefficiense*	BD	AP029120	Shiina *et al*., 2014
YM1-7	*B. diazoefficiense*	BD	AP029121	Shiina *et al*., 2014
YM2-2	*B. diazoefficiense*	BD	AP029122	Shiina *et al*., 2014
YM2-3	*B. diazoefficiense*	BD	AP029123	Shiina *et al*., 2014
YM2-5	*B. diazoefficiense*	BD	AP029124	Shiina *et al*., 2014
YM2-7	*B. diazoefficiense*	BD	AP029125	Shiina *et al*., 2014
YM3-2	*B. diazoefficiense*	BD	AP029126	Shiina *et al*., 2014
YM3-4	*B. diazoefficiense*	BD	AP029127	Shiina *et al*., 2014
HK1-31	*B. diazoefficiense*	BD	AP029128	Shiina *et al*., 2014
HK1-32	*B. diazoefficiense*	BD	AP029129	Shiina *et al*., 2014
HK2-8	*B. diazoefficiense*	BD	AP029130	Shiina *et al*., 2014
HK3-7	*B. diazoefficiense*	BD	AP029131	Shiina *et al*., 2014
HK3-13	*B. diazoefficiense*	BD	AP029132	Shiina *et al*., 2014
HK3-21	*B. diazoefficiense*	BD	AP029133	Shiina *et al*., 2014
HK3-25	*B. diazoefficiense*	BD	AP029134	Shiina *et al*., 2014
FK2-2	*B. diazoefficiense*	BD	AP029135	Shiina *et al*., 2014
FK2-3	*B. diazoefficiense*	BD	AP029136	Shiina *et al*., 2014
FK2-4	*B. diazoefficiense*	BD	AP029137	Shiina *et al*., 2014
FK2-6	*B. diazoefficiense*	BD	AP029138	Shiina *et al*., 2014
KM4-1	*B. diazoefficiense*	BD	AP029139 (AP029140)	Shiina *et al*., 2014
KM4-2	*B. diazoefficiense*	BD	AP029141 (AP029142, AP029143)	Shiina *et al*., 2014
MY1-2	*B. diazoefficiense*	BD	AP029144	Shiina *et al*., 2014
MY1-3	*B. diazoefficiense*	BD	AP029145	Shiina *et al*., 2014
MY1-4	*B. diazoefficiense*	BD	AP029146	Shiina *et al*., 2014
KG1-5	*B. diazoefficiense*	BD	AP029147 (AP029148)	Shiina *et al*., 2014
KG1-11	*B. diazoefficiense*	BD	AP029149	Shiina *et al*., 2014
KG1-13	*B. diazoefficiense*	BD	AP029150 (AP029151)	Shiina *et al*., 2014
KG2-5	*B. diazoefficiense*	BD	AP029152 (AP029153)	Shiina *et al*., 2014
KG2-6	*B. diazoefficiense*	BD	AP029154 (AP029155)	Shiina *et al*., 2014
KG2-73	*B. diazoefficiense*	BD	AP029156 (AP029157)	Shiina *et al*., 2014

[a]type of nosZ gene determined by *in silico* PCR using bradyrhizobial species-specific *nosZ* primer sets: BD, *B. diazoefficiense* type ; BW, *B. ottawaense*

[b]IDs of plasmid are shown in parentheses

Table S1-continued

Strains	Taxonomy	nosZ type[a]	Accession number[b]	Reference
D110				
A2C-35	*B. diazoefficiense*	BD	AP029061	This study
A2C-1	*B. diazoefficiense*	BD	BAAAAB010000000	This study
A2C-39	*B. diazoefficiense*	BD	AP029062	This study
A2C-2	*B. diazoefficiense*	BD	BAAAAC010000000	This study
A2C-24	*B. diazoefficiense*	BD	AP029063	This study
A2C-29	*B. diazoefficiense*	BD	AP029064	This study
A2C-30	*B. diazoefficiense*	BD	AP029065	This study
A2C-26	*B. diazoefficiense*	BD	AP029066	This study
A2C-12	*B. diazoefficiense*	BD	AP029067	This study
A2C-18	*B. diazoefficiense*	BD	AP029068 (AP029069)	This study
A2C-3	*B. diazoefficiense*	BD	AP029070	This study
A2C-13	*B. diazoefficiense*	BD	AP029071	This study
A2C-19	*B. diazoefficiense*	BD	AP029072	This study
A2C-17	*B. diazoefficiense*	BD	AP029073	This study
A2C-60	*B. diazoefficiense*	BD	AP029074	This study
A2C-61	*B. diazoefficiense*	BD	AP029075	This study
A2C-92	*B. diazoefficiense*	BD	AP029076	This study
A2C-5	*B. diazoefficiense*	BD	BAAAAD010000000	This study
A2C-6	*B. diazoefficiense*	BD	AP029077	This study
A2C-34	*B. diazoefficiense*	BD	AP029078	This study
A2C-54	*B. diazoefficiense*	BD	AP029079	This study
A2C-16	*B. diazoefficiense*	BD	AP029080	This study
A2C-37	*B. diazoefficiense*	BD	AP029081	This study
A2C-58	*B. diazoefficiense*	BD	AP029082	This study
A2C-51	*B. diazoefficiense*	BD	AP029083	This study
X110				
XF1	*B. diazoefficiense*	BD	AP023091.1	Minamisawa *et al*., unpublished
XF2	*B. diazoefficiense*	BD	AP023092.1	Minamisawa *et al*., unpublished
XF3	*B. diazoefficiense*	BD	AP023093.1	Minamisawa *et al*., unpublished
XF4	*B. diazoefficiense*	BD	AP023094.1	Minamisawa *et al*., unpublished
XF5	*B. diazoefficiense*	BD	AP023095.1	Minamisawa *et al*., unpublished
XF6	*B. diazoefficiense*	BD	AP023096.1	Minamisawa *et al.,* unpublished
XF7	*B. diazoefficiense*	BD	GCF_003183845.2	Minamisawa *et al*., unpublished
XF8	*B. diazoefficiense*	BD	AP023097.1	Minamisawa *et al*., unpublished
XF9	*B. diazoefficiense*	BD	AP023098.1	Minamisawa *et al*., unpublished
XF10	*B. diazoefficiense*	BD	AP023099.1	Minamisawa *et al*., unpublished
XF11	*B. diazoefficiense*	BD	AP023100.1	Minamisawa *et al*., unpublished
XF12	*B. diazoefficiense*	BD	AP023101.1	Minamisawa *et al*., unpublished
XF13	*B. diazoefficiense*	BD	AP023102.1	Minamisawa *et al*., unpublished
XF14	*B. diazoefficiense*	BD	AP023103.1	Minamisawa *et al*., unpublished
XF15	*B. diazoefficiense*	BD	AP023104.1	Minamisawa *et al*., unpublished
XF16	*B. diazoefficiense*	BD	AP023105.1	Minamisawa *et al*., unpublished
XF17	*B. diazoefficiense*	BD	AP023106.1	Minamisawa *et al*., unpublished
XF18	*B. diazoefficiense*	BD	AP023107.1	Minamisawa *et al*., unpublished
XF19	*B. diazoefficiense*	BD	AP023108.1	Minamisawa *et al*., unpublished
SG09				
SG09	*B. ottawaense*	BO	GCF_009176665.1	Wasai-Hara *et al.,* 2019
BWmix				
SG09	*B. ottawaense*	BO	GCF_009176665.1	Wasai-Hara *et al*., 2019
SG10	*B. ottawaense*	BO	BTIO00000000.1	Wasai-Hara *et al*., 2019
SG20	*B. ottawaense*	BO	BTIH00000000.1	Wasai-Hara *et al*., 2019
SG23	*B. ottawaense*	BO	BTII00000000.1	Wasai-Hara *et al*., 2019
SH12	*B. ottawaense*	BO	BTIJ00000000.1	Wasai-Hara *et al*., 2023
SH14	*B. ottawaense*	BO	BTIK00000000.1	Wasai-Hara *et al*., 2023
SH17	*B. ottawaense*	BO	BTIL00000000.1	Wasai-Hara *et al*., 2023
SH20	*B. ottawaense*	BO	BTIM00000000.1	Wasai-Hara *et al*., 2023
SI12	*B. ottawaense*	BO	BTIN00000000.1	Wasai-Hara *et al*., 2023
SI19	*B. ottawaense*	BO	BTIO00000000.1	Wasai-Hara *et al*., 2023
SI21	*B. ottawaense*	BO	BTIP00000000.1	Wasai-Hara *et al*., 2023
F1-1	*B. ottawaense*	BO	BAAAAE010000000	Minakata *et al*., 2023

[a]type of nosZ gene determined by *in sillico* PCR using bradyrhizobial species-specific *nosZ* primer sets: BD, *B. diazoefficiense* type ; BW, *B. ottawaense*

[b]IDs of plasmid are shown in parentheses

Table S2 Primer sets for nodule analysis and amplicon sequence

Target gene	Primer name	sequence (5' to 3')	References
**For nodule analysis**			
nosZ (*Bradyrhizobium diazoefficiens*)	nosZ_BD-F	GACGGCGATACCATGAAAGT	Itakura *et al*., 2013
	nosZ_BD-R	TCGGGTTGATCTTGGAACGG	This study
nosZ (*Bradyrhizobium ottawaense*)	nosZ_BW-F	CAAGCCCGAGAATGATCAGC	This study
	nosZ_BW-R	CCGGCCCTTCATCTCCATAT	This study
**For amplicon sequence**			
16S V3-V4	S-D-Bact-0341-b-S-17	CCTACGGGNGGCWGCAG	Klindworth *et al*., 2013
	S-D-Bact-0785-a-A-21	GACTACHVGGGTATCTAATCC	Klindworth *et al*., 2013
nosZ Clade I	nosZ1039F	GGCAARCTVTCDCCVAC	Zhang *et al*., 2021
	nosZ1393R	AVCGGTCYTTVGAGAAYTT	Zhang *et al*., 2021

Table S3. Summary of soil properties obtained in this study

Field	Sample name	pH[a] (H_2O)		total C[a] (%)		total N[a] (%)		C/N[a]	
Field A-1	Native-2020	5.73 ± 0.04	d	5.54 ± 0.05	f	0.40 ± 0.00	g	13.73 ± 0.03	f
	C110-2020	5.75 ± 0.05	d	5.49 ± 0.04	f	0.40 ± 0.00	g	13.81 ± 0.04	f
Field A-2	Native-2021	5.92 ± 0.13	cd	5.56 ± 0.07	f	0.41 ± 0.01	g	13.64 ± 0.12	f
	C110-2021	5.95 ± 0.13	cd	5.56 ± 0.03	f	0.41 ± 0.00	g	13.69 ± 0.07	f
	D110-2021	6.01 ± 0.14	cd	5.55 ± 0.06	f	0.41 ± 0.00	g	13.70 ± 0.09	f
	X110-2021	5.93 ± 0.13	cd	5.54 ± 0.03	f	0.40 ± 0.00	g	13.72 ± 0.06	f
	SG09-2021	5.93 ± 0.12	cd	5.59 ± 0.05	f	0.41 ± 0.00	g	13.65 ± 0.15	f
	BWmix-2021	5.92 ± 0.09	cd	5.59 ± 0.05	f	0.41 ± 0.00	g	13.75 ± 0.13	f
Field B-1	Till	6.13 ± 0.10	bcd	6.06 ± 0.24	ef	0.42 ± 0.01	fg	14.44 ± 0.66	ef
Field B-2	NoTill-O	6.66 ± 0.26	a	31.89 ± 2.22	c	1.73 ± 0.04	b	18.42 ± 1.15	c
	NoTill-A1	6.58 ± 0.05	ab	17.52 ± 1.86	d	1.09 ± 0.12	d	16.01 ± 0.49	d
	NoTill-A2	6.30 ± 0.29	abc	8.70 ± 0.38	ef	0.57 ± 0.03	efg	15.35 ± 0.23	de
Forest A	FA-O	5.96 ± 0.44	cd	41.86 ± 0.76	a	2.10 ± 0.07	a	19.98 ± 0.43	b
	FA-A1	4.89 ± 0.08	e	17.84 ± 0.64	d	1.16 ± 0.04	d	15.43 ± 0.89	de
	FA-A2	4.63 ± 0.08	ef	9.08 ± 1.04	ef	0.60 ± 0.07	ef	15.23 ± 0.92	de
Forest B	FB-O	6.01 ± 0.11	cd	37.02 ± 4.19	b	1.47 ± 0.18	c	25.22 ± 0.85	a
	FB-A1	5.07 ± 0.18	e	20.38 ± 3.30	d	1.22 ± 0.16	d	16.72 ± 0.56	d
	FB-A2	4.33 ± 0.05	f	9.92 ± 0.34	e	0.62 ± 0.01	e	15.97 ± 0.36	d

[a] Different letters indicate a significant difference among land management type ($p < 0.05$, Tukey's HSD test)

Table S4. Relative abundance of bacterial class among soil management types.

Taxonomy	Relative abundance (%)[a]								
	CC[b]			OC[b]			F[b]		
Acidobacteria Gp1	5.7 ±	1.5	a	0.3 ±	0.3	b	6.4 ±	3.7	a
Acidobacteria Gp2	1.8 ±	0.6	a	0.7 ±	0.9	b	7.2 ±	5.7	a
Acidobacteria Gp3	5.4 ±	1.5	a	1.3 ±	0.6	b	6 ±	3.7	a
Acidobacteria Gp6	2.1 ±	0.6	b	4.3 ±	2.0	a	1.7 ±	0.7	b
Actinobacteria	5.6 ±	0.9	n.s.	7.3 ±	2.9	n.s.	6.4 ±	3.8	n.s.
Flavobacteria	0.2 ±	0.1	b	4.9 ±	4.9	a	4.2 ±	5.8	a
Sphingobacteria	2.3 ±	0.6	b	6.3 ±	3.9	a	6.0 ±	3.7	a
Gemmatimonadetes	4.9 ±	0.5	a	1.4 ±	0.4	b	1.3 ±	0.7	b
Alphaproteobacteria	7.1 ±	0.9	b	12.7 ±	3.1	a	16.6 ±	4.8	a
Betaproteobacteria	7.5 ±	1.0	a	7.2 ±	0.5	ab	6.9 ±	2.9	b
Gammaproteobacteria	3.1 ±	0.7	b	6.6 ±	2.2	a	10.1 ±	5.2	a

[a] Different letters indicate a significant difference among land management type ($p < 0.05$, Steel-Dwass test.)
[b] Management type (Table 1). CC, conventional cropping; OC, organic cropping; F, Forest.
